# Neural correlates of own and close-other’s name recognition: ERP evidence

**DOI:** 10.3389/fnhum.2014.00194

**Published:** 2014-04-04

**Authors:** Pawel Tacikowski, Hanna B. Cygan, Anna Nowicka

**Affiliations:** ^1^Laboratory of Psychophysiology, Department of Neurophysiology, Nencki Institute of Experimental BiologyWarsaw, Poland; ^2^Brain, Body and Self Laboratory, Department of Neuroscience, Karolinska InstituteStockholm, Sweden

**Keywords:** self, close other, subject’s own name, person recognition, familiarity, personal relevance, ERP, P300

## Abstract

One’s own name seems to have a special status in the processing of incoming information. In event-related potential (ERP) studies this preferential status has mainly been associated with higher P300 to one’s own name than to other names. Some studies showed preferential responses to own name even for earlier ERP components. However, instead of just being self-specific, these effects could be related to the processing of any highly relevant and/or frequently encountered stimuli. If this is the case: (1) processing of other highly relevant and highly familiar names (e.g., names of friends, partners, siblings, etc.) should be associated with similar ERP responses as processing of one’s own name and (2) processing of own and close others’ names should result in larger amplitudes of early and late ERP components than processing of less relevant and less familiar names (e.g., names of famous people, names of strangers, etc.). To test this hypothesis we measured and analyzed ERPs from 62 scalp electrodes in 22 subjects. Subjects performed a speeded two-choice recognition task—familiar vs. unfamiliar—with one’s own name being treated as one of the familiar names. All stimuli were presented visually. We found that amplitudes of P200, N250 and P300 did not differ between one’s own and close-other’s names. Crucially, they were significantly larger to own and close-other’s names than to other names (unknown and famous for P300 and unknown for P200 and N250). Our findings suggest that preferential processing of one’s own name is due to its personal-relevance and/or familiarity factors. This pattern of results speaks for a common preference in processing of different kinds of socially relevant stimuli.

## Introduction

The occurrence of our own name usually signals that some potentially important information (a warning, a threat, a praise, etc.) will be directed to us. Because this happens countless times throughout a lifetime, people probably start to respond to this stimulus in a highly preferential and automatic manner. Many studies have confirmed the special status of own name processing.

For example, even 4–5 month-old infants prefer to listen to their own rather than other names (Mandel et al., 1995). The first lexical item that children learn to read and write is invariably their own name (Levin et al., 2005). Demented patients are able to recognize this specific stimulus, even when their perception of time and place is greatly deteriorated (Fishback, 1977). After general anesthesia, reactivity to subject’s own name precedes reactivity to pain or noise (Kurtz et al., 1977). Own name evokes behavioral responses even during sleep (Oswald et al., 1960) and results in awakening of the sleeping individuals (Portas et al., 2000). Own name has also been shown to have strong attention grabbing properties (Cherry, 1953; Moray, 1959; Wolford and Morrison, 1980; Wood and Cowan, 1995; Shapiro et al., 1997; Arnell et al., 1999; Conway et al., 2001; but see Harris and Pashler, 2004; Kawahara and Yamada, 2004).

Event-related potential (ERP) studies show strong modulation of brain activity by one’s own name. Specifically, they pointed mainly to the significance of the P300 component during processing of one’s own name (P300 is a positive component occurring around 300 ms after the stimulus onset, with its maximum over central-parietal scalp sites). P300 amplitude is larger for one’s own name than for other names (Berlad and Pratt, 1995; Müller and Kutas, 1996; Folmer and Yingling, 1997; Gray et al., 2004; Perrin et al., 2005; Zhao et al., 2009; Tacikowski and Nowicka, 2010; Tacikowski et al., 2011a; Fan et al., 2013; Cygan et al., 2014), especially if spoken by a familiar voice (Holeckova et al., 2006). Perrin et al. (1999) found differential P300 responses to own name even during sleep and Fischer et al. (2008) showed this effect in comatose patients.

P300 has been related to multiple cognitive functions, including context updating, allocation of attentional resources and associative memory processes during encoding and retrieval (for review see Polich, 2007). However, in the context of person recognition it has been associated mainly with the access to semantic information about the person whose name or face is being recognized. Support for this claim comes from observations that P300 is modulated by: (1) the familiarity of names and faces; (2) the number of repetitions during the study; and (3) the semantic priming. In contrast, it does not differentiate between names and faces (Schweinberger, 1996; Bentin and Deouell, 2000; Eimer, 2000; Paller et al., 2000; Tacikowski et al., 2011a).

Apart from P300, differential processing of self- vs. other names was shown for the N250 component (Zhao et al., 2009). N250 is a relatively small negative deflection occurring around 250 ms after stimulus presentation. Its maximum is found in temporal-parietal scalp sites. N250 amplitude is: (1) larger for familiar than for unfamiliar names and faces; (2) larger for perceptually primed than unprimed names and faces; and (3) unaffected by the semantic priming (Sommer et al., 1997; Pfütze et al., 2002; Schweinberger et al., 2002a; Pickering and Schweinberger, 2003). As a result, N250 was suggested to reflect the process of matching the input name or face to representations of names and faces stored in long-term memory (Miyakoshi et al., 2007).

Interestingly, Höller et al. (2011) showed that preferential processing of aurally presented own name could occur even around 150 ms after the stimulus presentation. Analogous evidence for visual presentation of one’s own name is missing. However, similar early effects (at the level of N170) were found for self-face processing (Keyes et al., 2010). N170 is a negative component occurring around 170 ms after stimulus presentation and it has its maximum in parietal-occipital scalp sites. N170 is often larger for names than for faces in the left hemisphere (Schweinberger et al., 2006; Tacikowski et al., 2011a) and larger for faces than for names in the right hemisphere (Rossion and Jacques, 2008). Moreover, N170 is rather unaffected by the familiarity of names and faces (Eimer, 2000; Rossion et al., 2000; Schweinberger et al., 2004) and/or priming manipulations (Pfütze et al., 2002; Schweinberger et al., 2002a,b, 2006). As a consequence, N170 is typically associated with stimulus-category discrimination.

Another early ERP component that showed self-preferential effects is the P200 component. It occurs approximately 200 ms after stimulus presentation and its maximum is over frontal-central scalp sites. Hu et al. (2011) reported that processing of semantic autobiographical information—participant’s full name, date of birth and hometown—was related to larger P200 responses than stranger’s full name and self-irrelevant date and place. Fan et al. (2013) found that P200 was larger to the name of the participant than to the name of participant’s father and to the names of famous people. Similar results were shown for processing of self- vs. other-relevant personality trait words (Mu and Han, 2010; Liu et al., 2013). The effects present for this component have been interpreted in terms of highly arousing and attention-grabbing nature of self-related information (Hu et al., 2011).

In sum, previous studies suggest that the processing of own name is preferential and that this preference occurs at the early (N170, N250 and P200) and late (P300) stages of information processing. The question that arises is whether these effects are own-name specific or common for other highly relevant and highly familiar social stimuli. If preferential processing of own name is due to its high adaptive value and high frequency of occurrence, a similar preference should be present for names of friends, family members, etc., because these names are also highly relevant and highly familiar.

On a more general level, the above issue relates to the structure of self-representation, or more specifically, to the extent to which self-representation is shared with representations of other people (Aron et al., 1991, 2004). Apart from being relevant to basic research, investigating the *self-other sharedness* seems to also be valid from the clinical perspective. People with Autism Spectrum Disorder (ASD) show atypical patterns of differentiating between self- and other-related information (Uddin et al., 2008; Cygan et al., 2014). Lombardo et al. (2010) proposed that ASD is related to difficulties in appreciating the similarities and differences between the self and other people, which results in theory-of-mind (ToM) deficits. Deficits in self- and other-mentalizing were also observed in schizophrenic patients (Langdon et al., 1997; Harrington et al., 2005).

It is noteworthy that previous findings do not enable to fully understand the role of relevance and familiarity factors in processing of one’s own name. This is because these studies did not manipulate both of these factors at the same time. In turn, investigating the above issue requires using at least four conditions: (1) own name, which is highly relevant, highly familiar and self-related; (2) close other’s name, which is highly relevant and highly familiar but not self-related; (3) famous person’s name, which is less relevant, less familiar and not self-related; and (4) unknown name, which is irrelevant, unfamiliar and not self-related. It is noteworthy that we use the term “self-related” in a very narrow sense, i.e., “designating the subject”, and not in the broad sense, i.e., “relevant to the subject”.

We assume that if the preference in processing of own name is due to high relevance and high familiarity factors and not solely due to its self-relatedness, the following pattern of results should occur: (1) both self- and close-other conditions will differ from famous and unknown conditions and (2) self- and close-other conditions will not differ from each other. To test this hypothesis thoroughly, we analyzed behavioral (accuracy rate and reaction time) and ERP measures in four different conditions: self, close other, famous and unknown. Apart from the above mentioned N170, P200, N250, and P300 components, we also included P100 in our analyses as it may serve as a marker of early stimulus-driven processing (Mangun, 1995; Luck et al., 2000).

## Materials and methods

### Participants

Twenty-two right-handed volunteers (12 male and 10 female) between the ages of 17–31 (mean = 23.3, SD = 4.6) participated in this study. None of them had ever changed their first or last name. Handedness was verified with the Edinburgh Inventory (Oldfield, 1971). All subjects were free from any neurological dysfunctions and had normal or corrected-to-normal vision. None of the subjects had any previous experience with the experimental task.

The Bioethics Committee of Warsaw Medical University approved the experimental protocol and informed consents were obtained from all the subjects prior to the study. The subjects were compensated for their participation.

### Stimuli

Similarly to our previous studies (Tacikowski and Nowicka, 2010; Tacikowski et al., 2011a,b, 2013; Cygan et al., 2014), we used participants’ full names (still called “names” for the ease of reference) instead of the first names only. This manipulation was actually necessary for the experimental task (i.e., to be able to discriminate between different types of “others”—see “Experimental Procedure” section below). Furthermore, a conjunction of the first and last name provides a more specific “label” of a given person than the first name or the last name only. Increasing this specificity was suitable for our research question.

All names were presented visually (white letters against a black background). The size of stimuli ranged from 2° × 2° to 2° × 6°, and did not differ between name-categories. There were four categories of names: (1) subject’s own name (50 presentations); (2) a name of a close-other (50 presentations); (3) a name of a famous person, e.g., a politician, actor, athlete, etc., (50 presentations); and (4) three unknown names (each presented 50 times, resulting in 150 presentations within this category). In order to make the task more engaging, each of the six names was written in five different fonts (e.g., Arial, Verdana, Times New Roman), in capital or regular letters, resulting in 10 different visual forms of each name. The font type and the size were fully matched between name-categories and between subjects.

Each set of stimuli was individually tailored. Different famous and unknown names were chosen for each subject to match for gender and length of the own and close-other’s names. Each set of stimuli consisted of the names of 3 women and 3 men. Before the experiment each participant was asked to confirm that he/she knew the famous name (“What is the profession of this person?”) and did not know the unknown names (“Do you know anybody whose name is … ?”). The mean lengths (in number of letters ± the standard deviation) of the first names were as follows: own (5.9 ± 1.5), close-other’s (6.1 ± 2.0), famous (5.8 ± 1.5) and unknown (5.9 ± 1.5). The mean lengths of the last names were as follows: own (9 ± 2.0), close-other’s (8.9 ± 3.1), famous (8.5 ± 1.5) and unknown (8.6 ± 2.1). The lengths of the names from different categories did not differ significantly.

Noteworthy, no restriction was placed on subjects’ choice of the close-other. By this, we wanted to avoid a situation in which a pre-defined person (e.g., a mother, a partner, etc.) is not really *close* to a particular subject. Instead, participants were simply asked to choose the most significant person in their life (7 participants chose their mother, 1 participant chose his father, 3 chose their siblings, 1 his grandmother, 1 her cousin, 2 their best friends, and 7 their partners).

### Experimental procedure

The stimuli were displayed in central vision on a 19-inch NEC MultiSync LCD 1990Fx monitor. For stimuli presentation and measurement of the subjects’ responses we used Presentation® software (Neurobehavioral Systems, Albany, CA, USA). The participants were seated in an acoustically and electrically shielded dark room at a distance of 60 cm from the computer monitor.

Although there were four categories of names, the subjects performed a two-choice recognition task: familiar (own, close-other’s, and famous person’s names) vs. unfamiliar (three unknown names). Subjects were to respond as quickly and as accurately as possible, by pressing one of two buttons on a Cedrus response pad (RB-830, San Pedro, USA). Participants used only the index and the third finger of the right hand to press the keys. The key assignment was counterbalanced on the group-level: half of the participants pressed the left key in response to familiar names and the right key in response to unfamiliar names while for the other half the reverse key assignment was used.

The number of presentations was adjusted to equalize the probability of each type of response (150 familiar and 150 unfamiliar names). The order of stimuli presentation was pseudo-randomized, so that no more than three names of the same category or three names written in the same font were presented consecutively. After reading instructions displayed on the computer screen, each session began with the participant completing a training session in which feedback information was displayed (“correct”, “incorrect”, or “response too slow”). During this session stimuli from each category were presented twice. After successful completion of this part, subjects began the actual study.

The sequence of events in each trial was as follows: presentation of a fixation point (a white “+” against a black background) for 100 ms, a blank screen for 500 ms, and a target item (a name) displayed for 500 ms. Next, the participants were shown a blank screen for 2000 ms and during this time they were to give a response. The inter trial interval (ITI) was randomly set to 100, 200 or 300 ms. The experiment lasted about 15 min.

### EEG recordings

EEG was continuously recorded from 62 scalp sites using a 136-channel amplifier (QuickAmp, Brain Products, Enschede, the Netherlands) and BrainVisionRecorder® software (Brain Products, Munich, Germany). Ag-AgCl electrodes were mounted on an elastic cap (ActiCAP, Munich, Germany) and positioned according to the extended 10–20 system. Electrode impedance was kept below 5 kΩ. The EEG signal was recorded against an average of all channels calculated by the amplifier hardware. Sampling rate was 500 Hz.

### Behavioral data analysis

Responses were scored as correct if the appropriate key was pressed within 150–2000 ms after the stimulus onset. Pressing the wrong key or pressing no key at all was treated as an incorrect response. Although subjects performed a speeded two-choice recognition task (familiar vs. unfamiliar) response times (RTs) and accuracy rates were analyzed for each name category separately (i.e., own, close-other, famous, unknown). For each participant, percentage accuracy and RTs were analyzed only for one unfamiliar name (randomly chosen from the set of all three unknown names). This was done to match the intra-experimental stimulus-familiarity factor (i.e., the number of repetitions during the experiment) between stimuli categories.

RTs and accuracy rates were analyzed using one-way repeated-measures ANOVA with “type of name” as a within-subject factor at four levels: own, close-other, famous and unknown. RTs were averaged across correct trials only. All effects with more than one degree of freedom in the numerator were adjusted for violations of sphericity according to the Greenhouse-Geisser formula (Greenhouse and Geisser, 1959). The results are reported with significance at *p* < 0.05.

### ERP analysis

Off-line analysis of the EEG was performed using BrainVisionAnalyzer® software (Brain Products, Gilching, Germany). The first step in data preprocessing was the correction of ocular artifacts using Independent Component Analysis (ICA; Bell and Sejnowski, 1995). After the decomposition of each data set into maximally statistically independent components based on visual inspection of the component map (Jung et al., 2001), the components representing eye blinks were rejected. Ocular-artifact-free EEG data were obtained by back-projecting the remaining ICA components after they were multiplied using the reduced component-mixing matrix. Butterworth zero phase filters were then implemented: high-pass −0.1 Hz, 12 dB/oct; low-pass −30 Hz, 12 dB/oct; and notch filter −50 Hz. Next, the EEG was segmented to obtain epochs extending from 200 ms before to 1000 ms after the stimulus onset (baseline correction from −200 to 0 ms). In the automatic artifact rejection procedure, the maximum permitted voltage step per sampling point was 50 µV, the maximum permitted absolute difference between two values in the segment was 200 µV, the minimum and maximum permitted amplitudes were −200 µV and 200 µV, and the lowest permitted activity in the 100 ms interval was 0.5 µV. ERPs were computed against the average reference.

ERPs for each name-category were computed for correct trials only (a special “macro” was run to select those epochs). Analogously to the behavioral data analysis, ERPs for the unknown condition were computed for only one of the unknown names. This was done to ensure that the intra-experimental stimulus familiarity and the signal-to-noise ratio were matched between stimuli categories. The mean number of segments in which subjects responded correctly and which passed the artifact rejection procedure was as follows: own name (46), close-other’s name (45), famous name (43), and unknown names (46). The number of epochs used to compute ERPs did not differ significantly between name categories. We analyzed ERP components that are commonly observed in person-recognition studies, i.e., P100, N170, N250 and P300 (Berlad and Pratt, 1995; Müller and Kutas, 1996; Folmer and Yingling, 1997; Schweinberger et al., 2002b, 2006; Gray et al., 2004; Perrin et al., 2005; Herzmann and Sommer, 2007; Zhao et al., 2009; Tacikowski and Nowicka, 2010; Tacikowski et al., 2011a; Cygan et al., 2014). Our analysis also included the P200 as some previous studies showed a self-preference for this component (Mu and Han, 2010; Hu et al., 2011; Fan et al., 2013).

The mean of values at each time point within a certain interval was used to assess the amplitudes of our ERP components of interest. This method is less affected by possible low signal-to-noise ratio than the peak measures methods (Luck, 2005). Based on the visual inspection of grand-average ERPs and based on the existing literature, the following time-windows were used: 80–120 ms after stimulus onset (P100), 130–220 ms (N170), 150–250 ms (P200), 220–320 ms (N250), and P300 (350–750 ms). Due to clear differences in the latencies of P300 to different conditions (Figure 2A), the 350–750 ms time window was subdivided into two time-windows: 350–550 ms and 550–750 ms.

**Figure 1:**
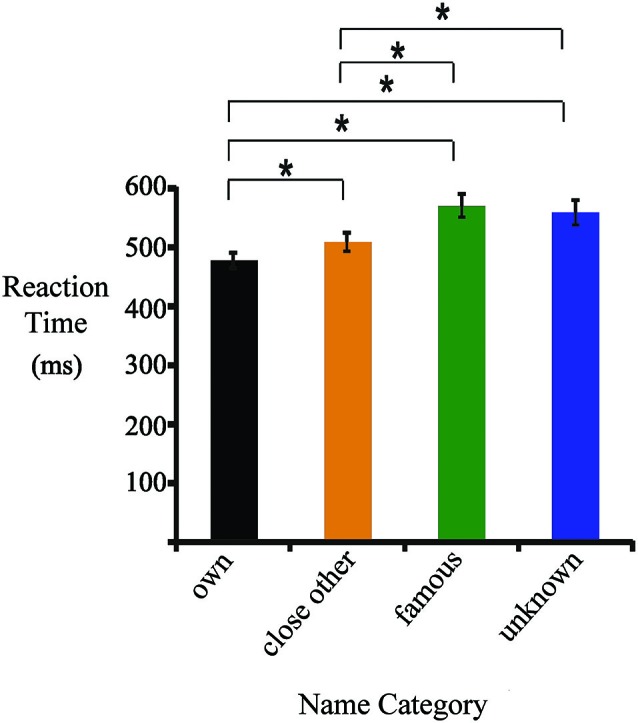
**Mean reaction times (RTs) for one’s own, close-other’s, famous and unknown names.** Significant differences are marked by asterisks.

**Figure 2:**
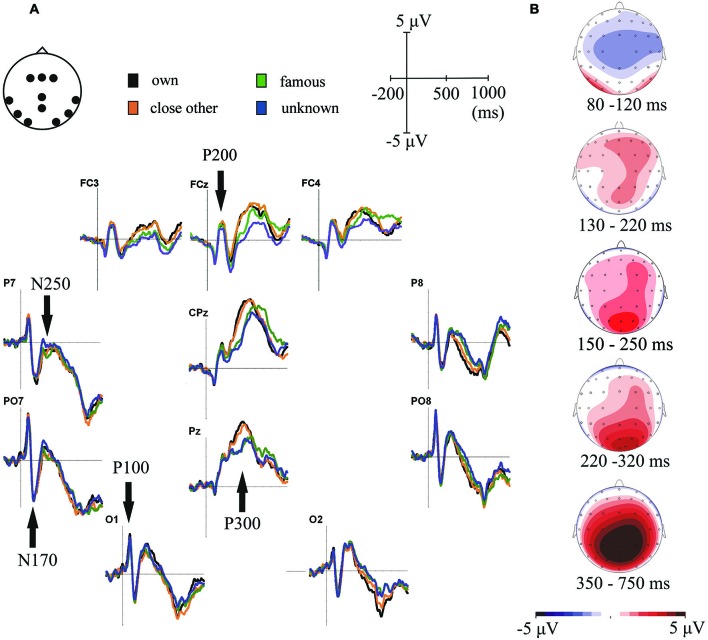
**(A)** Grand-average ERPs for one’s own, close-other’s, famous and unknown names. ERPs are presented for electrode locations chosen for statistical analyses. **(B)** Topographical distributions of the ERP components-of-interest computed for all experimental conditions averaged together.

In our analyses we focused on scalp regions in which the above-mentioned ERP components had their maximum amplitudes (see Figure 2B). These localizations were highly consistent with scalp distributions reported in previous studies (e.g., Schweinberger et al., 2002b, 2006; Herzmann and Sommer, 2007; Mu and Han, 2010; Tacikowski and Nowicka, 2010; Hu et al., 2011; Tacikowski et al., 2011a; Liu et al., 2013). P100 was analyzed in bilateral occipital sites (O1 and O2), P200 in frontal-central electrodes (FCz, FC3 and FC4), N170 and N250 in bilateral parietal-occipital electrodes (PO7 and PO8) and P300 in the central-parietal sites (CPz and Pz).

For each ERP component we performed a two-way repeated-measures ANOVA with the following within-subject factors: “type of name” (four levels: own, close-other, famous and unknown) and “electrode location”. The latter was at two levels for P100, N170, N250 (left vs. right), and P300 (anterior vs. posterior) and at three levels for P200 analysis (left vs. central vs. right). All effects with more than one degree of freedom in the numerator were adjusted for violations of sphericity according to the Greenhouse-Geisser formula (Greenhouse and Geisser, 1959). The results are reported with significance at *p* < 0.05.

## Results

### Behavioral results

The percentage accuracies of responses to all categories of names were as follows (mean percentage ± standard error): one’s own name (99 ± 1%), close-other’s name (98 ± 1%), famous name (92 ± 2%), and unknown names (98 ± 2%). Statistical analysis showed the main effect of “type of name” (*F*_3,19_ = 11.28, *p* = 0.001, $\eta_{p}^{2}=0.35$), with one’s own, close-other’s and unknown names being recognized better than famous names (*p* = 0.012; *p* = 0.005; and *p* = 0.022, respectively). The accuracy of responses to the first three types of names did not differ significantly between each other.

Figure 1 presents mean RTs (± standard error) for all categories of names. They were as follows: one’s own name (477 ± 13 ms), close-other’s name (510 ± 13 ms), famous name (570 ± 20 ms), and unknown name (561 ± 21 ms). ANOVA revealed a main effect of “type of name” (*F*_3,19_ = 40.92, *p* < 0.0001, $\eta_{p}^{2}=0.66$). *Post-hoc* tests showed that RTs to one’s own name were shorter than to close-other’s (*p* < 0.0001), famous (*p* < 0.0001) and unknown names (*p* < 0.0001). In addition, RTs to close-other’s name were shorter than to famous (*p* < 0.0001) and unknown names (*p* < 0.0001). Differences between RTs to famous and unknown names were not significant.

### Electrophysiological data

#### P100

Statistical analysis of this component showed only the main effect of “electrode location” (*F*_1, 21_ = 11.22, *p* = 0.003, $\eta_{p}^{2}=0.35$). Processing of all categories of names was associated with significantly larger P100 amplitudes in the left than in the right hemisphere. Figure 2A illustrates this result.

#### N170

Analysis of this component also revealed only the main effect of “electrode location” (*F*_1,21_ = 9.21, *p* = 0.006, $\eta_{p}^{2}=0.31$). Analogously to P100 results, all categories of names were associated with larger N170 amplitudes in the left than in the right parietal-occipital region (see Figure 2).

#### P200

The significant main effect of “type of name” was found for this component (*F*_3,19_ = 6.26, *p* = 0.001, $\eta_{p}^{2}=0.23$). *Post-hoc* analyses showed that self- and close-other’s names did not differ from each other, however, both of them were related to larger P200 amplitudes than unknown names (*p* = 0.033 and *p* = 0.003, respectively).

#### N250

ANOVA for this component showed main effects of “type of name” (*F*_3,19_ = 3.9, *p* = 0.013, $\eta_{p}^{2}=0.16$) and “electrode location” (*F*_1,21_ = 7.81, *p* = 0.011, $\eta_{p}^{2}=0.27$). The amplitude of N250 was larger in the left than in the right parietal-occipital region. In addition, amplitudes of N250 were larger for one’s own name than for unknown name (*p* = 0.029). An analogous effect was observed for close-other’s name, however, it was present only as a trend (*p* = 0.073). No significant effects were found for the famous name condition.

#### P300

Figure 2A shows P300 component in central-parietal scalp sites (i.e., CPz and Pz). Consistent with the visual inspection, ANOVA on P300 amplitudes in the earlier time window (350–550 ms) revealed the main effect of “type of name” (*F*_3,19_ = 5.49, *p* < 0.0001, $\eta_{p}^{2}=0.54$) and a significant “type of name” × “electrode location” interaction (*F*_3,19_ = 24.28, *p* = 0.002, $\eta_{p}^{2}=0.21$). *Post-hoc* tests showed that: (1) P300 amplitudes to one’s own name did not differ from P300 amplitudes to close-other’s name (*p* = 0.48); (2) P300 amplitudes to one’s own name were higher than to famous (*p* < 0.0001) and unknown names (*p* < 0.0001); (3) P300 amplitudes to close-other’s name were higher than to famous (*p* = 0.001) and unknown names (*p* < 0.0001); and (4) these effects were highly significant at both Pz and CPz electrodes, but were stronger for the former than for the latter. We did not find any significant effects in the later time window (550–750 ms).

#### Additional analyses

As mentioned before, the latency of P300 seemed to differ considerably between conditions (Figure 2A). To test this observation statistically we detected the peaks of P300 responses using global maxima search in the 350–750 ms time-window and then we entered those values to a 4 × 2 repeated-measures ANOVA, with “type of name” (self, close-other’s, famous, unknown) and “electrode location” (CPz vs. Pz) as the factors. We found the main effect of “type of name” (*F*_3,19_ = 8.56, *p* = 0.0001, $\eta_{p}^{2}=0.29$) and the main effect of “electrode location” (*F*_3,19_ = 5.62, *p* = 0.027, $\eta_{p}^{2}=0.21$). *Post-hoc* comparisons showed that P300 latencies: (1) were shorter to own name than to famous (*p* = 0.015) and unknown (*p* = 0.022) names; (2) were shorter to close-other’s name than to famous (*p* = 0.017) and unknown (*p* = 0.027) names; and (3) did not differ between self- and close-other’s names (*p* = 0.81). In addition, P300 responses were generally faster in the Pz than in the CPz electrode. Latencies of other ERP components were very similar across conditions, so we did not assess them in separate analyses (Figure 2A).

Because the amplitude and latency of P300 can be highly responsive to specific demands of a task (Johnson, 1988), we run additional correlation analyses to test whether these measures were somehow related to the speed of behavioral responses. The Pearson correlation method (two-tailed) did not show any significant effects.

## Discussion

The goal of this ERP study was to investigate whether enhanced ERP responses to own name are due to self-specific factors (i.e., the fact that the name designates the self), or maybe more generally, due to the high relevance and/or high familiarity of this stimulus. We assumed that, if the latter was the case, then: (1) own name and close-others’ name would be associated with similar preference in processing and (2) both own and close-other’s names would be related to larger amplitudes of N170, P200, N250, and P300 than less relevant and less familiar names, i.e., famous person’s and unknown names.

Behavioral data analysis showed that participants recognized their own name faster than all other names. Reaction times to close-other’s name were also shorter than to famous and unknown names. These results could not be attributed to different motor response requirements, as subjects pressed the same button for all familiar names, nor to the task-relevance factor, as subjects were instructed to differentiate only between familiar vs. unfamiliar names. Furthermore, because stimuli from each category were presented the same number of times, the effect also could not be explained by different intra-experimental familiarity factors. Instead, the above findings suggest an easy and to some extent automatic access to representations of highly familiar and emotionally salient social stimuli. This result is consistent with previous findings showing that self-preference occurs largely implicitly, even in the absence of any experimental task (e.g., Berlad and Pratt, 1995).

Analysis of the ERP data revealed some significant effects both for early (i.e., P100, N170, P200 and N250) and late (i.e., P300) ERP components. P100 and N170 responses were not modulated by the “type of name” factor, while P200, N250 and P300 components were.

The amplitude of P100 is typically modulated by physical attributes of stimuli, such as size, contrast and intensity (Coles and Rugg, 1995; for review see Rossion and Jacques, 2008). During person recognition tasks P100 is typically larger for faces than for names, which probably reflects the greater “perceptual richness” of the former (Pfütze et al., 2002; Tacikowski et al., 2011a). On the other hand P100 is not modulated by factors such as familiarity and self-relevance (e.g., Allison et al., 1999; Pfütze et al., 2002; Tacikowski et al., 2011a), which is in line with current findings.

With regard to N170, it is generally accepted that this component represents the structural analysis of a face (e.g., Eimer, 2000; Schweinberger et al., 2002a; Herzmann et al., 2004) or the word-form analysis of a name (Bentin et al., 1999). Both of these processes are pre-semantic, which explains why we did not find modulations of N170 by the familiarity and/or relevance factors.

In turn, we found that the amplitudes of P100 and N170 for all types of names were larger in the left than in the right hemisphere. Similar effects were reported also in previous studies (Pfütze et al., 2002; Schweinberger et al., 2006; Tacikowski et al., 2011a). These findings could be attributed to a typical dominance of the left hemisphere in language processing.

In our study the relevance and/or familiarity of names started to play a role around 200 ms after stimulus presentation. These effects were present for P200 and N250 components and became highly evident for the P300 component.

P200 amplitude was higher for self- and close-other conditions than for the unknown condition. This pattern of results is consistent with the hypothesis that P200 indexes automatic attention responses to highly arousing and highly attention-grabbing stimuli (Mu and Han, 2010; but see Liu et al., 2013). What is important for the aim of this study is that we did not find significant differences between P200 amplitudes for own and close-other’s names. It suggests the two are characterized by similarly arousing and attention-grabbing properties (Figure 2A).

With regard to N250, we found that its amplitude was larger for self- than for unknown names. A similar trend was observed for close-other’s name. N250 probably reflects the process of matching input name or face to the representations of names and faces stored in long-term memory (Herzmann and Sommer, 2007; Miyakoshi et al., 2007; Kaufmann et al., 2009; Tacikowski et al., 2011a). Our results suggest that this matching could be affected by the relevance and/or familiarity of the name being recognized. Crucially, we did not find any significant differences between N250 amplitudes to self and to close-other conditions, which suggests that the “matching” was similarly efficient for both of these names.

P300 amplitude did not differentiate between own and close-other’s names. However, both of these names were related to larger P300 amplitudes than famous and unknown names. Analogous pattern of results was present for the latencies of P300. This result is fully consistent with our hypothesis and suggests that P300 in response to own name is modulated by the relevance and/or the familiarity factors and not solely by the self-relatedness feature.

Enhanced P300 in response to one’s own name is in line with previous results (Müller and Kutas, 1996; Folmer and Yingling, 1997; Perrin et al., 2005; Tacikowski and Nowicka, 2010; Tacikowski et al., 2011a; Fan et al., 2013). Lack of significant differences between processing of one’s own and close-other’s names was also shown in fMRI studies (Sugiura et al., 2008; Tacikowski et al., 2013).

However, Fan et al. (2013) recently reported that own name was related to larger P300 than the name of the participant’s father. Analogous pattern was found also for the P200 component. We think that these inconsistencies are mainly due to the method of selecting close-others in two studies. In our study subjects chose a name of the most important person in their lives, whereas in the study by Fan et al. (2013) close-other was pre-selected by the experimenters (it was always the name of participant’s father). Without doubt many people consider their father to be a very important person in their lives. However, this is likely not true for everyone. Furthermore, the significance of parents generally decreases with age. As a consequence, our close-other was probably *closer-to-the-self* than the close-other used by Fan et al. (2013). This difference could explain why the comparison between self vs. close-other was non-significant in our study and reached significance in the study by Fan et al. (2013). Another methodological difference that could explain the above inconsistency is the use of implicit vs. explicit behavioral tasks. In Fan et al. (2013) study recognition of a name was not necessary, whereas in our experiment it was task-relevant. The effect of task-relevance on self-processing needs further investigation (see Cygan et al., 2014). Most importantly, both our and Fan et al. (2013) studies generally demonstrated the same pattern of results: the magnitude of self vs. close-other difference (as revealed by P300 amplitude) was reduced when compared to the magnitude of the self vs. distant other difference. Therefore, both studies suggest that the magnitude of self-preference is largely modulated by non-self specific factors, such as personal relevance.

In the context of person recognition, P300 has mainly been treated as an index of access to semantic memory (Paller et al., 2000; Schweinberger et al., 2002b, 2006; Herzmann and Sommer, 2007; Kaufmann et al., 2009; Tacikowski et al., 2011a). Our results are in line with this interpretation as both the self and close-other probably have much more elaborative and more complex semantic memory representations than famous and unknown people.

It has been shown that the amplitude of P300 also varies with the emotional value of stimuli. Emotionally charged stimuli, regardless of their valence, produce larger P300 then neutral stimuli (Johnston et al., 1986; Dietrich et al., 2001). According to Lang et al. (1997) model of motivated attention, emotional cues prompt motivational regulation and draw attentional resources. Some definitions characterize emotions as “psychophysiological states that reflect a person’s appraisal of the meaning, relevance, and value of events in the world” (Dolan, 2002). Being oblivious to own name could lead to missing some potentially important information directed to us, e.g., a threat, a warning, praise, etc. The same seems to hold for names of close-others—they are important for us. As a result, our findings for P300 could be explained by differences in motivational value, with subject’s own and close-other’s names being the most motivationally engaging, unknown names being the least and famous names being in between.

Alternatively, our P300 results could also be explained in terms of access to shared neural representation of the self and close-others (Aron et al., 1991, 2004; Gopnik and Meltzoff, 1994; Decety and Sommerville, 2003; Slotter and Gardner, 2009; Lombardo et al., 2010). Perceiving and appreciating the cognitive and emotional similarity between oneself and other people is necessary for the normal development of the self (Gopnik and Meltzoff, 1994; Decety and Sommerville, 2003). Self-other integration typically grows from the depth of shared experiences, which means that people with whom we have an emotional bond are more likely to be included into the concept of self than people who we are only acquainted with (Gopnik and Meltzoff, 1994; Slotter and Gardner, 2009).

In addition, it could not be ruled out that our P300 results were related to the frequency of occurrence factor. In everyday life people encounter their own and close-other’s names much more often than other names. Although previous studies showed that P300 component is modulated more by the semantic- than by the perceptual-familiarity factor (Schweinberger, 1996; Bentin and Deouell, 2000; Eimer, 2000; Paller et al., 2000; Tacikowski et al., 2011a), the issue needs further investigation.

One may suppose that the familiar vs. unfamiliar task itself could have attenuated the differences between processing of own, close-other and famous names (i.e., in this task they become exemplars of the same response category). However, this is rather unlikely as self-related processing is largely automatic. For example, Gray et al. (2004) showed that the P300 difference between self and other was present even if participants were focusing on detecting colored targets, and Berlad and Pratt (1995) reported larger P300 responses to own name even in the absence of an experimental task. In turn, using a familiar vs. unfamiliar discrimination task eliminated the unspecific variance due to different motor requirements and possibly reduced the bias created by the explicit task set. Nevertheless, the differences between implicit vs. explicit processing of self-related information require further research (Cygan et al., 2014).

Our analyses would probably benefit from including the subjective familiarity and relevance scores acquired for each stimulus name. For example, such data would enable us to test whether familiarity and relevance of famous names parametrically reduces the magnitude of self-preference. In addition, collecting such scores would allow us to better control our variables of interest, as it cannot be ruled out that we selected highly familiar and highly relevant famous others (e.g., a favorite actor) for some participants by accident. Future studies should collect such scores. However, the consistency between our and previous findings suggests that even if familiarity and relevance of famous names were not fully controlled, the effect was probably cancelled-out when analyzing the group data and did not substantially affect our general findings.

Finally, it needs to be mentioned that there was some dissociation between our RTs and ERP results. Subjects’ responses were significantly faster to own name than to close-other’s name but the difference between the two was non-significant for all the ERP components. It is noteworthy that all ERP components that we analyzed probably reflected the preparation of motor response, whereas the final RTs depended on both preparatory and execution processes. As a result, the discrepancy between behavioral and ERP data suggests that some self-specific preference could be present at the execution stage, but not at the preparation stage. This issue, however, needs further investigation.

Altogether our study shows that the magnitude of self vs. other difference in information processing is largely modulated by the relevance and/or familiarity factors (for a review see Symons and Johnson, 1997). The study also shows that the other is not a nominal variable but is instead a type of continuum that includes strangers, neighbors, co-workers, family, friends, etc. Future research can focus on disturbances of this self-other continuum in clinical populations, e.g., schizophrenia and autism.

## Conflict of interest statement

The authors declare that the research was conducted in the absence of any commercial or financial relationships that could be construed as a potential conflict of interest.
